# Ketogenic metabolic therapy for schizoaffective disorder: a retrospective case series of psychotic symptom remission and mood recovery

**DOI:** 10.3389/fnut.2025.1506304

**Published:** 2025-02-07

**Authors:** Nicole Laurent, Erin L. Bellamy, Katherine A. Tague, Donika Hristova, Ally Houston

**Affiliations:** ^1^Family Renewal, Inc., Vancouver, WA, United States; ^2^School of Psychology, University of East London, London, United Kingdom; ^3^Well Supported Behavioral Health, PLLC, Greenwich, CT, United States; ^4^Transformation Evoked, Virginia, MN, United States; ^5^Department of Psychiatry, University of Oxford, Oxford, United Kingdom

**Keywords:** ketogenic diet, ketogenic metabolic therapy, schizoaffective disorder, psychiatric recovery, case series, metabolic psychiatry, ketosis

## Abstract

**Background:**

Schizoaffective disorder is a severe psychiatric condition characterized by mood disturbances and psychotic symptoms. Standard treatments, primarily pharmacological, often fail to control symptoms fully and can lead to significant metabolic side effects. Emerging evidence suggests that ketogenic metabolic therapy (KMT), also known as the ketogenic diet, may offer a powerful alternative to conventional treatments for mood components and resolve psychiatric symptoms in patients with schizoaffective disorder.

**Methods:**

This case series investigates the effects of KMT on two individuals diagnosed with schizoaffective disorder who pursued this therapy due to the ineffectiveness of conventional treatments. Both case presentations followed a modified ketogenic diet with medical oversight. Symptom changes in mood were assessed using validated tools, including the Generalized Anxiety Disorder-7 (GAD-7), Depression Anxiety Stress Scales (DASS-42), PTSD Checklist for DSM-5 (PCL-5), and Patient Health Questionnaire-9 (PHQ-9).

**Results:**

Both case presentations experienced the complete cessation of psychotic symptoms and improvements in mood. Case 1, a 17-year-old female, achieved full remission of severe suicidal ideation, hallucinations, and anxiety within 6 weeks, with sustained improvements at a 24-week follow-up. Case 2, a 32-year-old female, achieved full remission of chronic psychotic and mood symptoms by 6 months. Patients either achieved full psychiatric deprescription or were in the process of deprescription at time of follow-up.

**Conclusion:**

This case series demonstrates that ketogenic metabolic therapy can resolve chronic psychotic and mood symptoms in patients with schizoaffective disorder, leading to full remission and significant functional recovery and reported improvements in quality of life that extend beyond symptom control with standard of care interventions.

## 1 Introduction

Schizoaffective disorder is a serious mental condition, characterized by both psychosis and mood disturbances (1). At present, the treatment for this condition relies on psychopharmacological interventions. Unfortunately, commonly prescribed medications may put patients at increased risk for weight gain, diabetes, hyperlipidemia, and cardiovascular disease (2). Indeed, psychosis itself has been associated with poor metabolic health (3, 4), a reality exacerbated by antipsychotic medications (5). These metabolic abnormalities, including insulin resistance and hyperglycemia, are not only seen in individuals treated with antipsychotics but have also been observed in treatment-naïve patients, highlighting a broader connection between psychosis and metabolic dysfunction (6, 7). In recent years, a growing body of evidence has suggested the presence of a relationship between psychosis and brain glucose hypometabolism (8–12). Brain glucose hypometabolism has been broadly linked to bioenergetic disruptions in psychotic disorders, contributing to symptoms and supporting the exploration of therapies that target energy metabolism (13).

Given these findings, Ketogenic Metabolic Therapy (KMT) has emerged as a potential treatment that may address both metabolic dysfunction and psychiatric symptoms (14). This approach may alleviate schizoaffective disorder through improved glucose metabolism, among other mechanisms (15). This dietary pattern may be particularly advantageous given its proven ability to improve psychotic (16–18) and mood cycling symptomologies (18–20) in prior studies.

To explore these potential therapeutic effects in the present case series, standardized mental health assessments were employed. An overview of the quantitative mental health assessment tools used in this case series can be seen in Supplementary Table 1.

## 2 Case presentation 1

### 2.1 Clinical and dietary background

A 17-year-old female with schizoaffective disorder experienced severe psychiatric symptoms, including persistent suicidal thoughts, auditory and visual hallucinations, and depression for 6 years, resistant to conventional treatments. Her symptoms, including anxiety and depression, began in middle school and worsened, leading to severe episodes requiring prolonged 24-h family supervision, periodic hospitalizations, and mood and psychotic symptoms that left her unable to pursue academics or work. At the time of diet implementation, she was on Olanzapine, Buspirone, Bupropion, and Fluoxetine, with prior psychotherapy and medications providing little improvement. After a recent hospitalization, she decided to try the ketogenic diet after it was researched by a family member. Her psychotherapist, general practitioner, and psychiatrist were informed and agreed to provide medical oversight. Only one team member was trained in ketogenic diet implementation and initiated the diet.

Supplementation included a methylated B-complex, trace minerals (zinc, copper, manganese, chromium, molybdenum, boron, iodine, and vanadyl sulfate), vitamin D with K2, and electrolytes (sodium, magnesium, and potassium). Additionally, 500 mg of Acetyl-L-Carnitine was prescribed daily due to low red meat intake prior to the diet and the lack of a timely carnitine level assessment. No adjustments were made to the supplements during the intervention. Before ketogenic therapy initiation, her diet consisted primarily of chicken nuggets, French fries, and pizza, accompanied by sugary beverages like sweet tea and soda. While her diet included protein sources like chicken, shrimp, and salmon, it was primarily centered around high-carbohydrate and high-sugar options, with minimal fresh, whole foods intake. Upon leaving inpatient treatment, the patient and her family immediately initiated some lower carbohydrate options, making it difficult to establish a true baseline for carbohydrate consumption. Starting biometrics included height at 5 feet 6 inches (168 cm), weight 82 kg (180 pounds), and body mass index (BMI) of 29. At final assessment weight was 77 kg (170 pounds) and BMI was at 27.4.

### 2.2 Ketogenic metabolic therapy intervention strategy

The trained team member provided basic education on diet composition, food groups, and monitoring tools as part of diet initiation. No adjustments were made to the supplements during the intervention. Macronutrient tracking was initiated using the Cronometer app, and dietary assessment was conducted before assigning ketogenic macros, indicating carbohydrate consumption was between 60 and 174 g per day.

Macronutrient ratios started at 2.5:1 for the first month (198 g fat, 56 g protein, 22 g net carbs) to prioritize ketone production and shifted to 2:1 (186 g fat, 70 g protein, 20 g net carbs) during treatment, both classified as modified ketogenic diets. The diet included beef, chicken, salmon, shrimp, eggs, and dairy, with fats mainly from dairy, butter, and avocado oil. Low-carb vegetables and homemade ketogenic desserts helped with compliance. KMT support began with 30-min virtual sessions twice a week for 12-weeks, then moved to once biweekly and then monthly for an approximate 6-month duration.

**Figure 1:**
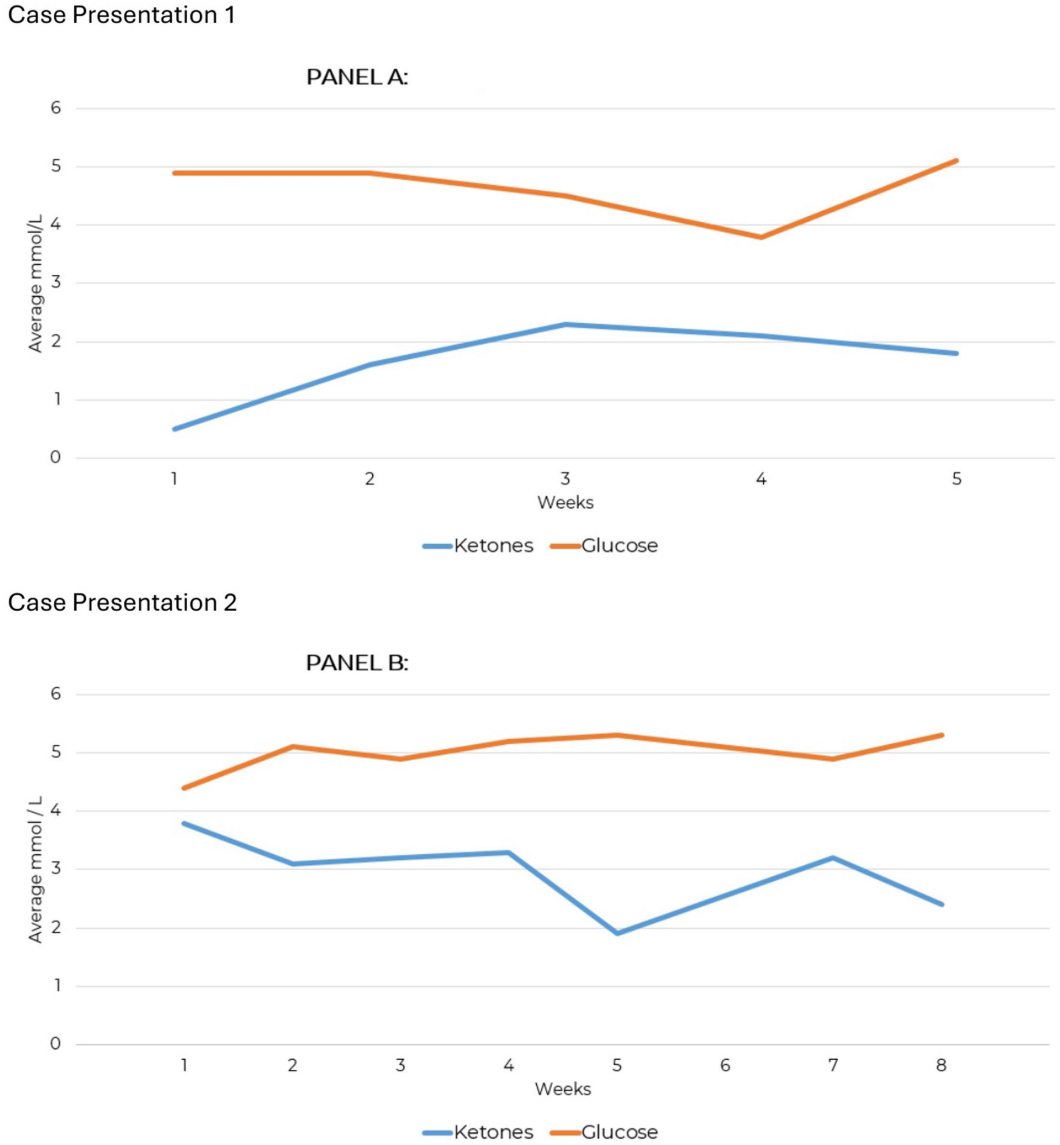
Weekly average ketone and measures for case presentations 1 and 2. **(A)** Average glucose/ketone levels. **(B)** Average glucose/ketone levels.

Blood glucose and beta-hydroxybutyrate (BHB) were tracked using a Keto-Mojo® GK+ Blood Glucose and β-Ketone Dual Monitoring System; nutritional ketosis was confirmed at 0.8 mmol/L. Testing compliance over 10 weeks total was 63% for daily ketones and 27% for daily glucose. The highest recorded BHB level was 3.5 mmol/L. Weekly averages for BHB and glucose are shown in Figure 1A for the first 5 weeks, during which testing was consistent; subsequent testing was less consistent but still contributes to the overall compliance percentages.

### 2.3 Evaluation of intervention outcomes

After starting the ketogenic diet, the patient reported that her auditory hallucinations had ceased, and her severe psychiatric symptoms were described by her treatment team as being in remission. Her parents corroborated this finding. By 6 weeks, she reported that her suicidal ideation, depression, anxiety, and hallucinations had fully resolved. The Generalized Anxiety Disorder-7 (GAD-7), Depression Anxiety Stress Scales (DASS-42), and PTSD Checklist for DSM-5 (PCL-5) were assessed over a 24-week period (Supplementary Table 2).

**Figure 2:**
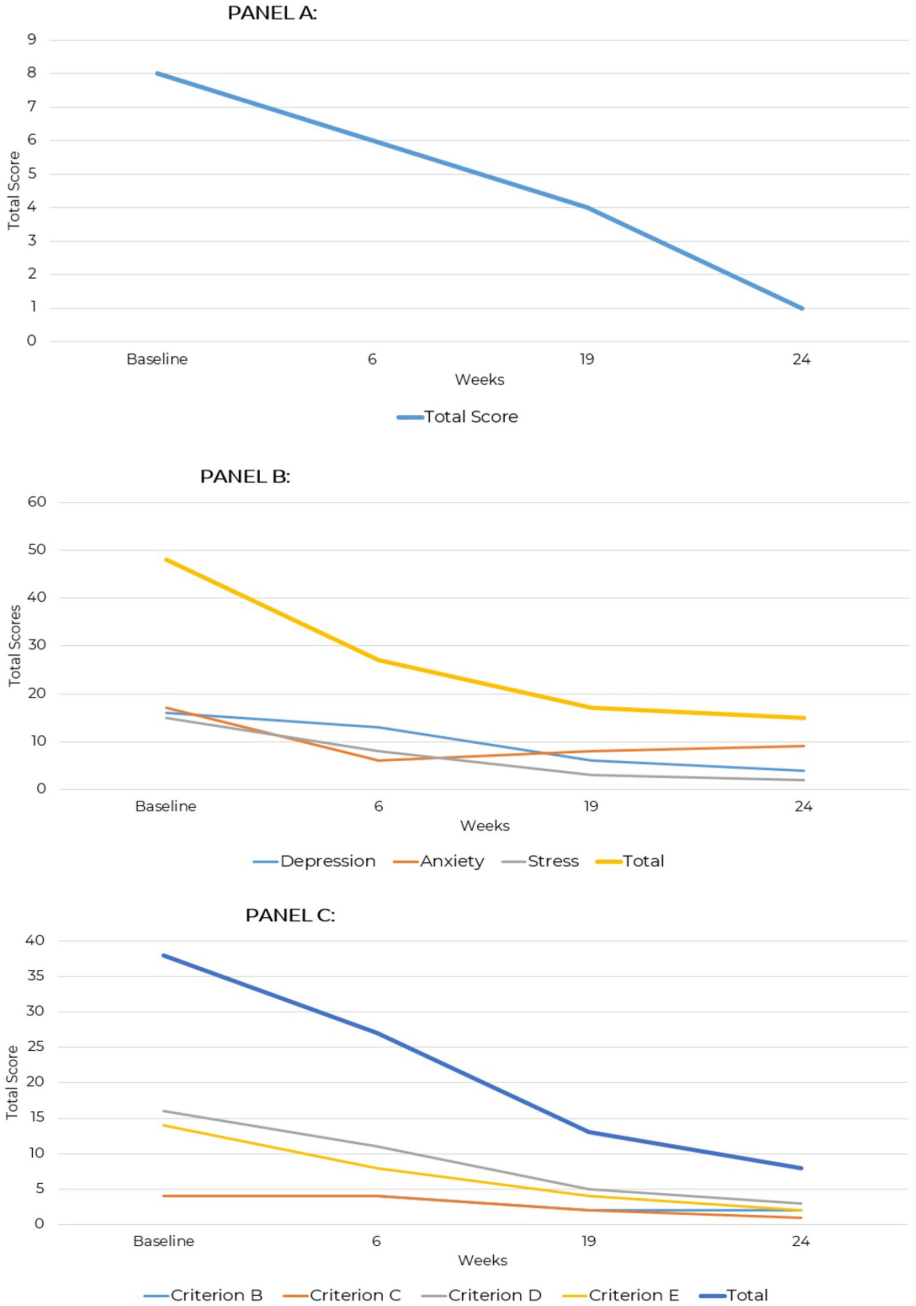
GAD-7, DASS-42, and PCL-5 total scores across two assessment points over a 24-week period for case presentation 1. **(A)** Generalized anxiety disorder (GAD-7). **(B)** Depression anxiety stress scale (DASS-42). **(C)** PTSD checklist for DSM-5 (PCL-5).

At baseline, the GAD-7 score indicated moderate-to-moderately severe anxiety, which had normalized by the time of follow-up, indicating the early treatment effects were sustained over time (Figure 2A). Baseline DASS-42 scores indicated moderate depression, mild anxiety, and moderate stress. By the 24-week follow-up, improvements were reported across all areas. The reduction to a score of 4 on the Depression subscale, given the patient’s psychiatric history of recurrent suicidal ideation requiring multiple hospitalizations, is of clinical note and importance. The stress subscale, which was 15 considered mild severity at baseline, had decreased to 2 by follow-up into normal range. Anxiety, initially moderate with a score of 17, improved to 9 within the mild range at follow-up (Figure 2B).

The patient’s baseline PCL-5 score of 38, indicating significant emotional distress characterized by hyperarousal, negative mood, and avoidance behaviors, likely reflected overlapping symptoms of mood disorders such as depression or anxiety, including negative cognitions, irritability, and mood dysregulation; this total score decreased to 8 at the 24-week assessment, reflecting a substantial reduction in symptoms. Criterion B (Intrusive Thoughts) and Criterion C (Avoidance) symptom endorsement reduced from 4 to 2 and 1. Criterion D (Negative Alterations in Cognition and Mood) showed the most improvement with a baseline score of 16 that reduced to 3, suggesting mood stabilization and fewer negative thoughts. Criterion E (Alterations in Arousal and Reactivity) declined from 14 to 2, indicating self-reported relief from hypervigilance, irritability, and sleep disturbances (Figure 2C).

It is relevant that the patient began the ketogenic diet shortly after being released from psychiatric hospitalization and was on stabilizing doses of psychotropic medication during baseline mood assessments. Therefore, the scores may not reflect the level of distress prior to hospitalization. By follow-up, the patient had reduced and eliminated all medications except Fluoxetine and was actively tapering under prescriber guidance, indicating a level of stability not previously achieved with medication and therapy alone. Additionally, the death of the patient’s childhood pet 2 weeks before reassessment may have influenced the follow-up scores.

She successfully returned to normal daily activities, including pursuing studies and working. Notably, she reports planning to attend college—a possibility that was not previously considered by her or her parents due to the severity of her symptoms. The family reports experiencing increased joy and strengthened relationships, attributing these positive changes to the patient’s implementation of ketogenic metabolic therapy as a treatment for schizoaffective disorder.

## 3 Case presentation 2

### 3.1 Clinical background

A 32-year-old female diagnosed with schizoaffective disorder at age 25 presented with severe psychiatric symptoms, including psychotic episodes that involved paranoid thoughts and auditory and visual hallucinations, cognitive impairment, and multiple suicide attempts, one requiring life support. Her illness began in high school with depressive symptoms and academic decline, resulting in multiple hospitalizations and a recommendation for lifelong institutionalization. At the time of diet implementation, she was on Lurasidone for psychosis and Metformin for PCOS, with limited symptom control and significant side effects. Her treatment history included a wide range of medications, including antipsychotics, mood stabilizers, antidepressants, anticonvulsants, electroconvulsive therapy (ECT), psychotherapy, and lifestyle changes like sleep hygiene and exercise, all with limited success. Comorbidities included PCOS and pituitary microadenoma. She was open to trying the ketogenic diet specifically to address her psychiatric symptoms. Her treatment team included one ketogenic-informed nutritional practitioner as part of a pre-established treatment team consisting of a primary care physician, a psychiatrist, and a psychotherapist.

### 3.2 Ketogenic metabolic therapy intervention strategy

Supplementation included a multivitamin with methylated B-complex, trace minerals (iodine, zinc, selenium, manganese, chromium, molybdenum, boron), vitamin D, K2, and A. Electrolytes were supplemented as sodium, magnesium bis-glycinate, and potassium citrate. Acetyl-L-carnitine was prescribed prophylactically at 1,000 mg in divided daily doses.

For the first 12 weeks, KMT support involved 30-min virtual meetings twice a week, transitioning to weekly and bi-weekly intervals later. Macronutrient tracking using the Cronometer app showed a baseline carbohydrate intake of 130–180 g per day. Before starting the diet, she often skipped breakfast, had a lunch of vegetables and protein, and ate carbohydrate-heavy dinners like pizza, pasta, quesadillas, and rice bowls. Snacks included nuts, crackers, cheese, and chocolate, with 1–2 cups of coffee and occasional alcoholic drinks. Her carbohydrate intake was gradually reduced over 2 weeks to 20 g net per day.

Testing compliance was 93% for daily ketone measures and 79% for daily glucose over an 8-week period. Blood glucose and beta-hydroxybutyrate (BHB) tracking showed that nutritional ketosis was achieved at 3.3 mmol/L (Figure 1). Afterward, glucose was measured sporadically, and a continuous ketone monitor (CKM) tracked BHB levels (Supplementary Figure 1). Ketone production was maintained consistently over 32 weeks, demonstrating significant adherence to KMT.

Approximately 17 weeks after diet implementation, the patient followed up with her physician for carnitine assessment due to reduced ketone levels. The results showed free carnitine at 22 μmol/L, indicating hypocarnitinemia, leading to an increase in acetyl-L-carnitine supplementation to 2,500 mg daily in divided doses.

Macronutrient ratios were initially set at 1.75:1 and adjusted to 1.5:1 with consistent production of ketone production between 1.0–4.0 mmol/L. The ratio was later adjusted to 2:1 ratio (190 g fat, 75 g protein, 20 g net carbohydrates) with ad libitum feeding within ketogenic ratios to support athletic activities and maintain normal BMI.

The diet consisted primarily of beef, chicken, eggs, and dairy, with primary fat sources including avocado oil-based mayonnaise, olive oil, macadamia nuts, MCT oil, and butter. Low-carbohydrate vegetables were regularly consumed, along with purchased keto treats sweetened with stevia, erythritol, and monk fruit.

### 3.3 Evaluation of intervention outcomes

The patient reported noticing a significant reduction in psychotic symptoms around 2 weeks into the intervention, though they reported that they believed the improvements likely began earlier. The reported symptoms progressively diminished, with psychotic symptoms resolving first, followed by self-reported improvements in mood and cognitive function.

GAD-7 scores consistently declined over time. At baseline, the total score was 6, indicating mild severity. By week 8, the score dropped to 2, representing minimal severity, and by week 13, it was 1. By week 22, the score reached 0, indicating no symptoms, and this absence of symptoms persisted through weeks 27 and 52.

Over the evaluation period, total DASS-42 scores steadily decreased, reflecting reduced symptom severity. At baseline, the total score was 15 indicating mild severity, with anxiety being the most elevated subscale at 7. By week 8, the total score dropped to 9 as depression resolved and anxiety decreased to 5. At week 13, the total score further declined to 5, with all subscales remaining in the normal range. By week 22, the total score was 4, and by week 27, all subscale scores were 0, indicating no symptoms. At week 52, the total score was 3, with anxiety slightly elevated at 1 and stress at 2, both remaining within the normal range. These results demonstrate consistent improvement and stabilization of symptoms over 52 weeks.

**Figure 3:**
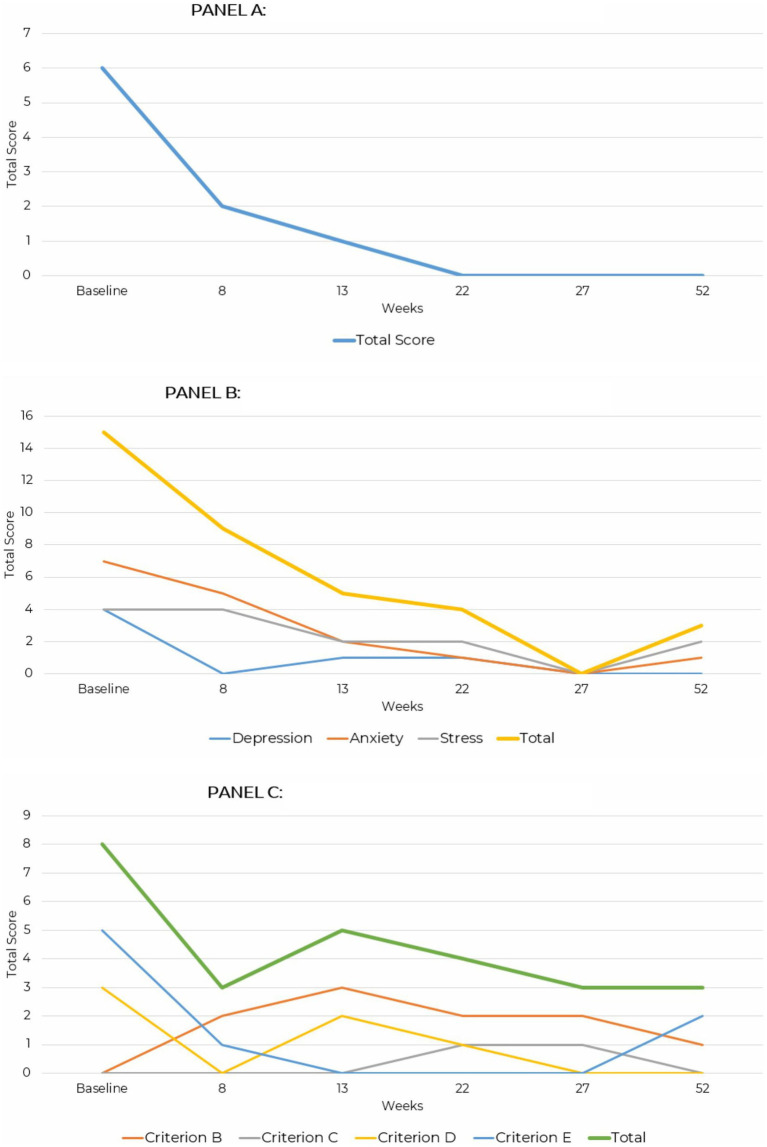
Line graph depicting the reduction in GAD-7, DASS-42, and PCL-5 and PHQ-9 total scores across assessment points over a 52-week period for case presentation 2. **(A)** Generalized anxiety disorder (GAD-7). **(B)** Depression anxiety stress scale (DASS-42). **(C)** PTSD checklist for DSM-5 (PCL-5).

PCL-5 scores showed fluctuations but an overall reduction in symptoms over time. At baseline, the total score was 8, driven by higher endorsement of Criterion E, reporting arousal and reactivity symptoms, and Criterion D with a score of 3, reflecting negative mood and cognition. By week 8, the score decreased to 3 as arousal and mood symptoms improved. At week 13, the score increased to 5 due to a rise in intrusion symptoms under Criterion B and negative mood under Criterion D. By week 22, the score declined to 4, with mild intrusion symptoms and minor mood disturbances. At week 27, the score remained at 3, indicating minimal symptoms. This score remained stable at week 52 reflecting only minor intrusion and arousal symptoms, with no clinically significant distress (Figure 3).

**Figure 4:**
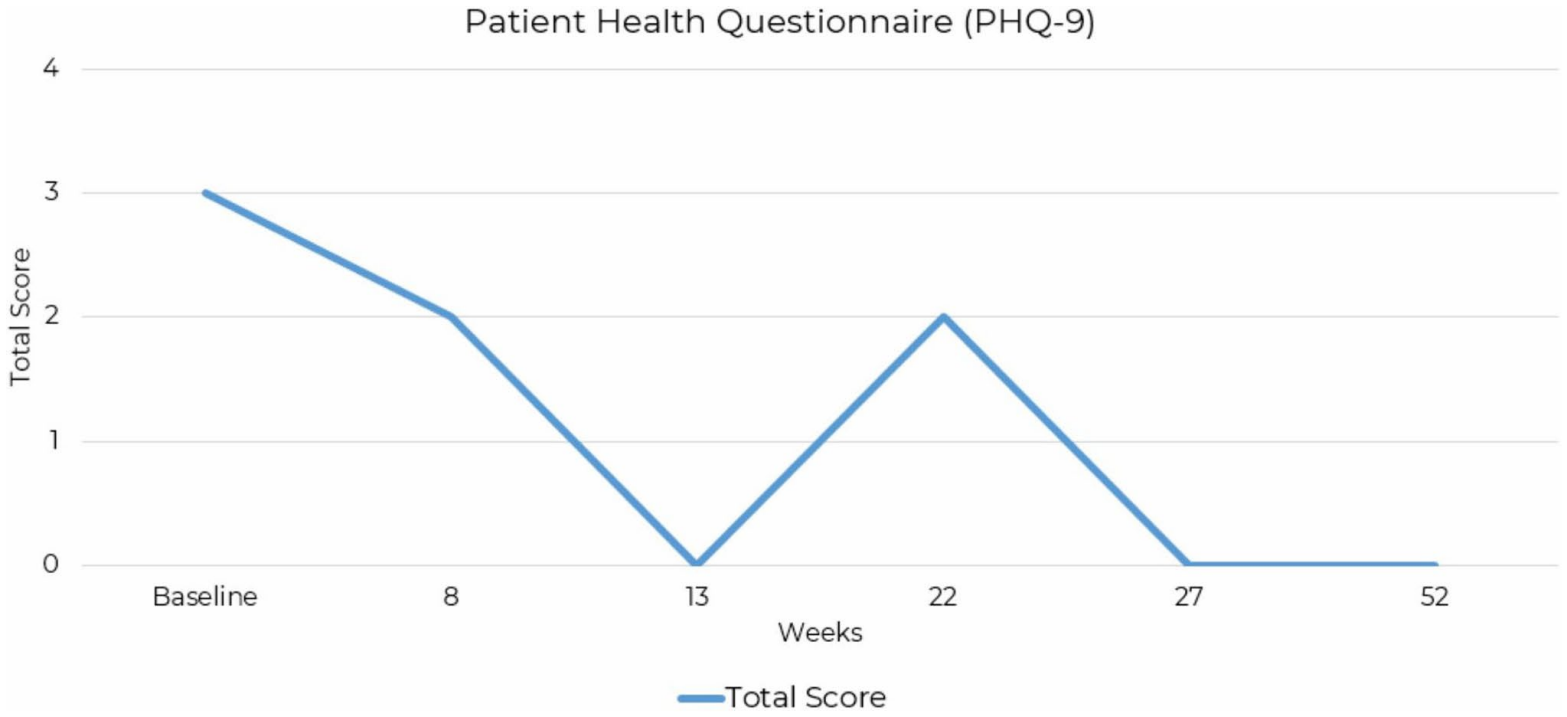
Line graph depicting the reduction PHQ-9 total scores across assessment points over a 52-week period for case presentation 2.

The Patient Health Questionnaire-9 (PHQ-9) measures the severity of depressive symptoms. A baseline score of 3 indicated minimal-to-no depressive symptoms endorsed, reducing slightly to a score of 2 at 8 weeks. By 13 weeks, the score dropped to 0, and temporarily increased to 2 before dropping to 0 at 27 weeks and maintaining a score of 0 at final assessment completed at 52-weeks (Figure 4).

Mood assessment severity levels reported are consistent with patient reports that negative symptoms, cognitive symptoms, hallucinations, and delusional thinking had a far greater impact on their daily life than mood-related symptoms at baseline. Although the PCL-5 scores showed a minor increase between weeks 8 and 13, attributed to a significant life stressor occurring over a 5-month period, the small magnitude of this fluctuation suggests potential resilience, as similar stressors had previously resulted in hospitalization. By week 22, the symptoms had decreased again, indicating that overall improvement was maintained despite the presence of a prolonged external stressor. At the time of follow-up, the patient had eliminated metformin and had successfully titrated off of Lurasidone completely under psychiatric care without relapse or increases in symptoms (Supplementary Table 3).

Alongside these psychiatric improvements, physical health markers were reflected over the course of treatment. At the start of treatment biomarkers included a height of 5 feet 7 inches (170 cm) and 125 lbs. (56.7 kg). The BMI remained stable with improved body composition as confirmed by bioelectrical impedance analysis (BIA). Measures showed a total BMI increase from 19.6 to 19.8. Body fat percentage dropped from 17.5 to 14.6%, with a 1.996 kg increase in skeletal muscle mass from Baseline through Week 27.

When asked about their experience using KMT, the patient replied, “It’s hard to think about how my life may have played out differently had I been offered this intervention earlier in the course of navigating my illness. I absolutely wish ketogenic therapy and metabolic therapies would have been shared with me as a viable option as early as possible.”

## 4 Discussion and conclusion

This case series presents two instances where KMT showed therapeutic effects against schizoaffective disorder, providing clinically relevant details of macronutrient ratios, supplementation, dietary management, biomarkers, and mood assessment. The frequency and duration of testing was not uniform across case presentations and reflected clinical flexibility in accommodating different levels of engagement with self-monitoring tools. The case presentations are retrospective and observational, designed to explore potential patterns without inferring causation between variables. Graphical presentations were intentionally constructed to reflect the independent nature of the data and avoid implying unsupported relationships.

In the first case, a 17-year-old female with treatment-resistant schizoaffective disorder showed rapid clinical improvements within 6 weeks of initiating a ketogenic diet and achieving ketosis. While previous treatments had failed to provide sustained relief, KMT led to a resolution in auditory hallucinations and persistent suicidal ideation. Standardized assessments revealed marked reductions in anxiety and depression, indicating a rapid therapeutic effect associated with the dietary intervention. Mild and transient elevations in mood assessments may be confounded by active medication tapering and temporary stressors from life events. The successful tapering of psychotropic medications, without a return of symptoms, suggests that KMT may have played a central role in the stabilization and recovery of this patient. A further limitation is that for these patients, no standardized measures of psychotic symptoms were utilized in their care and were therefore not reported.

In the second case, a 32-year-old female with a prolonged history of treatment-resistant schizoaffective disorder experienced a complete cessation in psychotic symptoms and improvements in mood and cognitive function shortly after starting KMT. Mood assessments demonstrated a steady reduction in both anxiety and depression, allowing for the gradual and complete tapering of psychotropic medications. Her schizoaffective disorder, which had persisted for over a decade, entered full remission with continued dietary adherence.

This case series differs from previous reports in that neither patient had obesity, and both intentionally pursued KMT for schizoaffective disorder. This distinction underscores the need for further research on KMT across diverse BMI categories and psychiatric profiles. Additionally, this series utilized a broader range of standardized mood scales, capturing improvements beyond diagnosis-specific psychotic symptoms across multiple domains of mood assessment. Further research is needed to establish standardized protocols for implementing KMT in psychiatric care.

This case series suggests that ketogenic metabolic therapy (KMT) may provide significant therapeutic benefits for patients with schizoaffective disorder, particularly in cases of treatment resistance. It contributes to the metabolic psychiatry literature by documenting symptom remission and sustained recovery over several months through consistent KMT adherence, following the failure of standard treatments. The sustained improvements observed in both cases emphasize the need for further research into KMT as a therapeutic intervention for complex psychiatric conditions such as schizoaffective disorder.

Future research should incorporate both quantitative measures and qualitative data, as seen in this case series. While quantitative measures reflected some changes in symptomatology, they did not fully capture the extent of recovery experienced by these two patients; therefore future studies should provide a comprehensive explanation of the potential of this treatment modality for psychiatric recovery. KMT Larger controlled studies are needed to further assess its potential generalizability and long-term efficacy.

## Data Availability

The original contributions presented in the study are included in the article/Supplementary material, further inquiries can be directed to the corresponding author.
