# Supplementary figures and images for: Ketogenic metabolic therapy for schizoaffective disorder: a retrospective case series of psychotic symptom remission and mood recovery
Source: Front Nutr. 2025 Feb 7;12:1506304. doi: 10.3389/fnut.2025.1506304 (PMC11844221; doi:10.3389/fnut.2025.1506304)

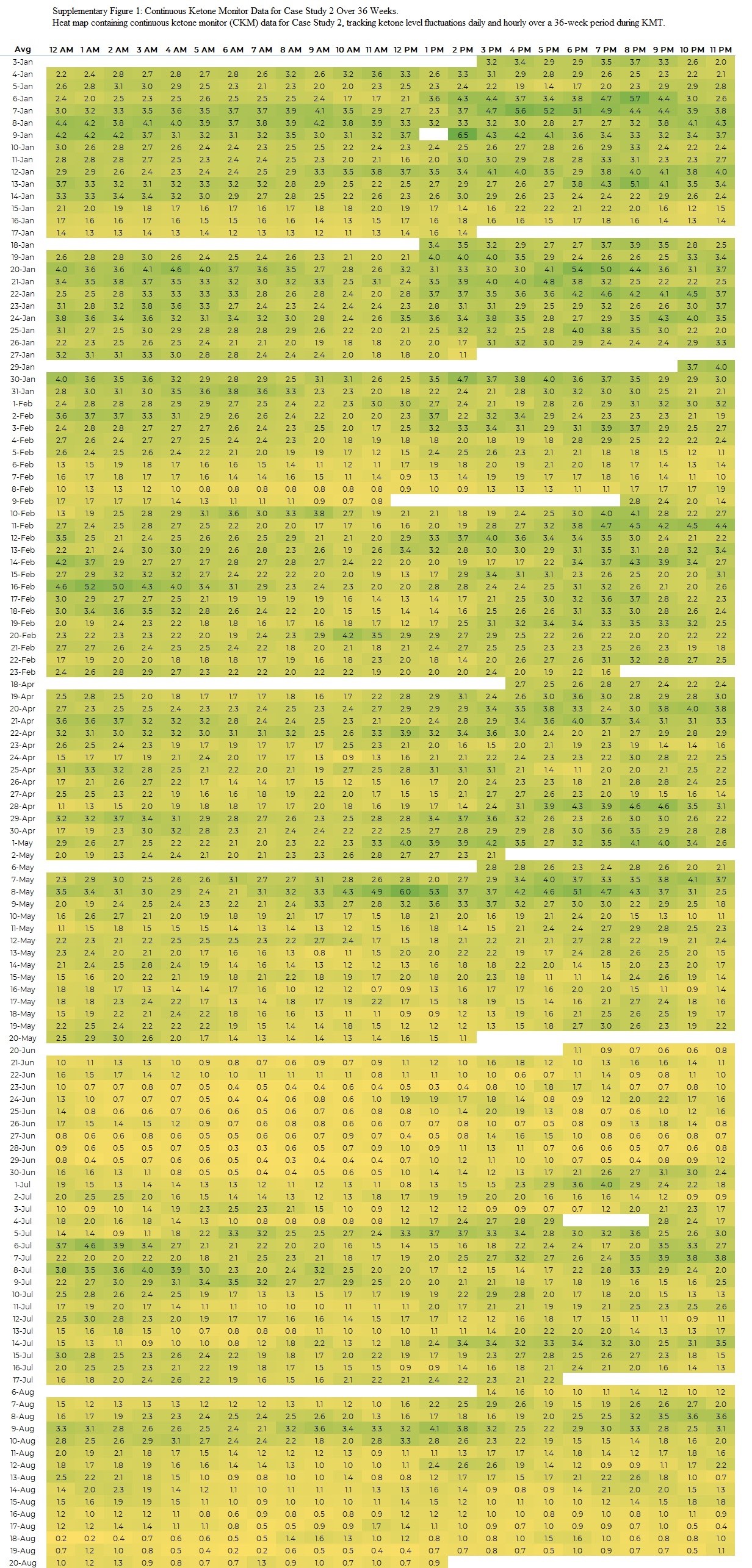

Supplement: Supplementary file 1 [file Image_1.jpeg]
