# Supplementary material for: Ketogenic metabolic therapy for schizoaffective disorder: a retrospective case series of psychotic symptom remission and mood recovery
Source: Front Nutr. 2025 Feb 7;12:1506304. doi: 10.3389/fnut.2025.1506304 (PMC11844221; doi:10.3389/fnut.2025.1506304)
Supplement: Supplementary file 2 [file Table_1.docx]

Supplementary Table 1. Overview of quantitative mental health assessment tools used in case series

| Assessment | What it Measures | Consistency | Reliability | Validity | Cut-Off Scores |
| --- | --- | --- | --- | --- | --- |
| GAD-7 (21) | The severity and presence of Generalized Anxiety Disorder symptoms | Strong Internal consistency (α = .92). | Strong test-retest reliability (ICC = 0.83). | Strong construct validity. | Anxiety Severity:  0-4: Minimal  5-9: Mild  10-14: Moderate  15-21: Severe |
| DASS-42 (22,23) | The presence of depression, anxiety and stress symptoms. | Strong internal consistency (α = .84-.91) | Satisfactory test-retest reliability  0.71 to 0.81 | Strong discriminant and concurrent validity. | Severity of Depression, Anxiety, Stress (respectively)  Normal: 0-9, 0-7, 0-14  Mild: 10-13, 8-9, 15-18  Moderate: 14-20, 10-14, 19-25  Severe: 21-27, 15-19, 26-33  Extremely Severe: 28+, 20+, 34+ |
| PCL-5 (24,25) | The presence of symptoms of PTSD, according to DSM-5 criteria | Strong internal consistency (α = .94 - .95) | Strong test-retest reliability (*r =* .89) | Strong convergent and divergent validity. | Presence of PTSD Symptomology  31-32*  **The PCL-5 was not originally intended to provide a severity score but as an assessment that evaluates the clinical criteria met in an individual* |
| PHQ-9  (26-28) | The presence and severity of depression symptoms. | Good internal consistency (α =*0.839)* | Strong test-retest reliability (ICC = 0.84) | Strong convergent and construct validity. | Depression Severity:  1-4: None  5-9: Mild  10-14: Moderate  15-19: Moderately Severe  20-27: Severe |
