# Supplementary material for: Ketogenic metabolic therapy for schizoaffective disorder: a retrospective case series of psychotic symptom remission and mood recovery
Source: Front Nutr. 2025 Feb 7;12:1506304. doi: 10.3389/fnut.2025.1506304 (PMC11844221; doi:10.3389/fnut.2025.1506304)
Supplement: Supplementary file 3 [file Table_2.docx]

|  | Case Presentation 1 | |
| --- | --- | --- |
| Assessment | Baseline | 24 Weeks |
| GAD-7 | 8 | 1 |
| DASS-42 | 48 | 15 |
| Depression Subscale | 16 | 4 |
| Anxiety Subscale | 17 | 9 |
| Stress Subscale | 15 | 2 |
| PCL-5 | 38 | 8 |
| Criterion B | 4 | 2 |
| Criterion C | 4 | 1 |
| Criterion D | 16 | 3 |
| Criterion E | 14 | 2 |
| PHQ-9 | N/A | N/A |
