# Supplementary material for: Ketogenic metabolic therapy for schizoaffective disorder: a retrospective case series of psychotic symptom remission and mood recovery
Source: Front Nutr. 2025 Feb 7;12:1506304. doi: 10.3389/fnut.2025.1506304 (PMC11844221; doi:10.3389/fnut.2025.1506304)
Supplement: Supplementary file 4 [file Table_3.docx]

|  | Case Presentation 2 | | | | | |
| --- | --- | --- | --- | --- | --- | --- |
| Assessment | Baseline | 8 Weeks | 13 Weeks | 22 Weeks | 27 Weeks | 52 Weeks |
| GAD-7 | 6 | 2 | 1 | 0 | 0 | 0 |
| DASS-42 | 15 | 9 | 5 | 4 | 0 | 3 |
| Depression Subscale | 4 | 0 | 1 | 1 | 0 | 0 |
| Anxiety Subscale | 7 | 5 | 2 | 1 | 0 | 1 |
| Stress Subscale | 4 | 4 | 2 | 2 | 0 | 2 |
| PCL-5 | 8 | 3 | 5 | 4 | 3 | 3 |
| Criterion B | 0 | 2 | 3 | 2 | 2 | 1 |
| Criterion C | 0 | 0 | 0 | 1 | 1 | 0 |
| Criterion D | 3 | 0 | 2 | 1 | 0 | 0 |
| Criterion E | 5 | 1 | 0 | 0 | 0 | 2 |
| PHQ-9 | 3 | 2 | 0 | 2 | 0 | 0 |
